# Consumer Information for clients with diabetes – a survey of Podiatrists current practice in the Hume region

**DOI:** 10.1186/1757-1146-8-S2-P13

**Published:** 2015-09-22

**Authors:** Louine Robinson

**Affiliations:** 1 Beechworth Health Service, Beechworth, Victoria, 3747, Australia

**Keywords:** Consumer information, health literacy, diabetes, podiatrists

## Background

This survey was undertaken as part of the work of the Consumer Information Working Party within the Hume Diabetes Service Improvement Collaborative HDSIC. The HDSIC is part of the implementation of the National Chronic Disease Strategy. The task of the HDSIC is to apply the National Diabetes Service Improvement Framework in the Hume region to increase the access to high quality diabetes service.

The objective of this survey was to understand podiatrists' decision-making and behaviour regarding consumer information given to clients with diabetes. This would identify gaps between current practice and Priority 4 *Provide clear and consistent information for people with chronic conditions and their carers* (Hume Regions Chronic Care Strategy HRCCS 2012-2022). This information will then be used by the collaborative to develop consumer information recommendations and support service providers to address issues relating to health literacy.

## Process

A total of 30 podiatrists from the public, private and education sector responded to an emailed a survey monkey questionnaire. It was designed to investigate clinicians' attitudes, source of information, selection criteria and any existing policies with regard to consumer information. Responses were collated and used to inform the work of the collaborative.

## Findings

With the exception of the two public health services in Hume there were no existing policies relating to the selection/provision of consumer information. The main sources of consumer information were Australian Podiatry Council and Diabetes Australia. Health Literacy was identified as the single most important factor in determining what consumer information was used with pictorial information rating highly.

## Conclusions

Podiatrists in the Hume Region have little formalised policy documentation regarding consumer information for clients with diabetes. They choose information from peak bodies and look for information, which is appropriate for their client's health literacy level. A possible area of improvement in health literacy is in the tailoring of information with regards to clients' cultural diversity.

